# Idiopathic Hirsutism and Insulin Resistance

**DOI:** 10.1155/2013/593197

**Published:** 2013-10-20

**Authors:** Afsaneh Talaei, Zahra Adgi, Mahnaz Mohamadi Kelishadi

**Affiliations:** ^1^Thyroid Disorders Research Center, Arak University of Medical Sciences, Arak 38481769401, Iran; ^2^Amir Almomenin Hospital, Arak 38481769401, Iran; ^3^Arak University of Medical Sciences, Arak, Iran

## Abstract

*Background and Objectives*. Polycystic ovary syndrome (PCOS) and idiopathic hirsutism (HI) are the two most common causes of hirsutism. Insulin resistance plays a key role in PCOS, but there are not enough data showing that patients with HI also have insulin resistance. This study was designed to evaluate the presence of insulin resistance in women with HI. *Methods*. Based on a cross-sectional study, two groups of age-BMI matched, hirsute women were compared to age-BMI matched, nonhirsute women. Sixty nonobese women with PCOS, thirty nonobese women with HI, and sixty nonobese control women were included in the study. Samples of hormones including androgens were measured. Insulin resistance based on homeostasis model assessment of insulin resistance (HOMA-IR) was compared between three groups by the Kruskal-Wallis test. *Results*. Patients with PCOS had significantly higher basal insulin level (16.04 ± 1.4 versus 7.32 ± 6.85 **μ**Iu/mL) and HOMA-IR score (3.7 ± 3.36 versus 1.75 ± 1.67) than patients with HI (*P*  0.001). Patients with HI also had significantly higher basal insulin level and HOMA-IR score than control group (*P*  0.001). *Conclusion*. Our data suggest that both PCOS and HI are associated with insulin resistance and these patients are more insulin resistant than healthy control people.

## 1. Introduction 

 Hirsutism, the presence of terminal hairs in females in a male-like pattern, is a common clinical condition which affects 5–10% of women of reproductive age. Hirsutism is extremely distressing for patients and has a significant negative impact on their psychosocial development [1]. Polycystic ovary syndrome (PCOS) and idiopathic hirsutism (HI) are the two most common causes of hirsutism [2].

PCOS is the most common endocrinopathy in women which is characterized by hyperandrogenemia and chronic anovulation [3]. Women with PCOS demonstrate marked clinical heterogeneity; the commonly associated features such as hirsutism, acne, polycystic-appearing ovaries, obesity, and acanthosis nigricans are neither uniform nor universal [4].

Insulin resistance and compensated hyperinsulinemia are the most features of PCOS [5]. According to some studies, the prevalence of diabetes, hypertension, hyperlipidemia, and cardiovascular disease is much more in PCOS patients than in general population [6].

Regarding the role of insulin resistance in PCOS, insulin resistance-lowering drugs are usually used to manage PCOS [7–9]. Rotterdam criteria have been generally used to describe PCOS, based on the exclusion of other androgen excess disorders and the presence of any two of oligoovulation or anovulation, clinical and/or biochemical hyperandrogenism, and polycystic ovaries on ultrasonography [10].

HI, the second most common cause of hirsutism after PCOS, is considered when hirsutism is associated with normal ovulatory function and normal circulating serum androgen concentrations. The pathogenesis of HI is still unclear, although increased activity of peripheral 5-alpha reductase enzyme [11], androgen receptor gene polymorphism [12], and increased sensitivity of hair follicles to androgens have been proposed [13].

It is well known that insulin resistance and hyperinsulinemia are closely associated with PCOS, and insulin-sensitizer drugs improve hirsutism in patients with PCOS, while there are not enough data showing that patients with HI also have insulin resistance [14].

The purpose of this study was to assess the presence/absence of insulin resistance in women with HI.

## 2. Methods

This cross-sectional study was performed in 60 hirsute patients with PCOS, 30 with HI, and 60 healthy women as control group between ages 16 and 40 years. The subjects were matched for age and BMI. The cases of PCOS and HI were selected from the patients who were referred to the endocrinology clinic, because of hirsutism. The study was approved by the local ethics committee. The protocol of the study was explained to all the subjects. Informed consent was obtained before the beginning of the study.

Hirsutism was defined according to the modified Ferriman-Gallwey (MFG) score >8. In this scoring system, 9 different body sites (upper lip, chin, chest, upper back, lower back, upper abdomen, lower abdomen, arm, and thigh) were scored. In each of these areas, a score of 0 (absence of terminal hairs) to 4 (extensive terminal hair growth) was assigned [15].

A standard questionnaire including demographic data and reproductive history, menstrual status, marital status, hyperandrogenism symptoms (hirsutism, acne, and hair loss), galactorrhea, family history of irregular menstrual cycle, and hirsutism was completed, during face-to-face interview. Anthropometric measurements, including waist, height, weight, and body mass index (BMI), were evaluated. Transabdominal ovary ultrasonography was performed for all subjects in days 3–9 of the menstrual cycle.

Blood fasting samples were collected to measure serum levels of free testosterone (FT), total testosterone (TT), dehydroepiandrosterone sulfate (DHEA-S), androstenedione, 17-hydroxy progesterone (17-OHP), and enzyme immunoassay (EIA) (Diagnostic Biochem Canada Co. Ontario, Canada). Prolactin, cortisol, follicle-stimulating hormone (FSH), luteinizing hormone (LH), thyroid-stimulating hormone (TSH), tetraiodothyronine (T4), immunoradiometric assay (IRMA) (Izotope, Budapest, Hungary), and insulin (electrochemiluminescence immunoassay (Roche diagnostics, Mannheim, Germany) were measured.

Serum levels of triglyceride (TG), total cholesterol (TC), high-density lipoprotein (HDL), low-density lipoprotein (LDL), and fasting plasma glucose (FPG) were determined by standard methods. Insulin resistance was calculated using homeostasis model assessment of insulin resistance (HOMA-IR) following formula [16]:

(1) $$\begin{array}{c} \text{HOMA-IR}=\frac{\text{FPG}\left(\text{mmol}/\text{L}\right)\times \text{Insulin}\left(\mu \text{U}/\text{mL}\right)}{22.5}. \end{array}$$

Thirty hirsute patients who had regular ovulatory cycles (luteal-phase progesterone levels greater than 4 ng/mL) and normal androgen levels were classified as HI group. 

The diagnosis of PCOS was made according to the Rotterdam 2003 criteria [10]. Sixty hirsute patients with PCOS had oligomenorrhea (bleeding at intervals of greater than 35 days) amenorrhea (absence of menstruation for 6 months), or polycystic ovarian morphology on ultrasonography (the presence of 12 or more follicles in each ovary measuring 2–8 mm in diameter and/or increased ovarian volume >10 mL) and clinical or biochemical hyperandrogenism [10] determined by laboratory measurements (greater than normal androgen levels based on the reference values of the commercial kits).

None of the control subjects had ovarian dysfunction or clinical or biochemical hyperandrogenism. None of the subjects had received any drugs known to interfere with hormonal levels for at least 3 months before the study. Cushing syndrome, nonclassical congenital adrenal hyperplasia, thyroid disorders, hyperprolactinemia, and androgen-secreting tumors were excluded by appropriate tests as previously described.

Statistical analysis was performed by Statistics Package for Social Science (SPSS version 16.0, SPSS Inc., Chicago, IL, USA). The results are presented as mean ± SD (standard deviation). The categorical variables, expressed as percentages, were compared using the chi-square test. The variables were assessed for normality using Kolmogorov-Smirnov test (K-S test). Levene's test was used to evaluate equality of variances before further statistical analysis.

 We used one-way ANOVA and Kruskal-Wallis test for comparison of variables within three groups in parametric and nonparametric conditions, respectively. Comparison between two groups was made by independent sample *t*-test or Mann Whitney *U* test in parametric and non parametric conditions, respectively. Laboratorial and clinical data were compared between groups using analysis of covariance (ANCOVA) to control for BMI.

Logistic regression was also used to adjust the effects of confounding variables for multivariate analysis. *P* value less than 0.05 was considered statistically significant. 

## 3. Results 

We studied 60 patients with PCOS, 30 patients with HI, and 60 healthy subjects as control group. All subjects in three groups did not differ in mean age and BMI (Table 1). Clinical parameters were similar between PCOS and HI (Table 2). The MFG score was also similar between both groups (12.7 ± 1.5, 11.6 ± 2), respectively, (*P* 0.9).

Lipid profile was not significantly different between PCOS, HI, and control groups (Table 3). Although insulin level and HOMA-IR were significantly higher in patients with PCOS and HI than in control subjects (*P* 0.001), they were significantly lower in HI group than in PCOS group (*P* 0.001). The other hormonal profile was not significantly different between all groups (Table 4).

## 4. Discussion

The purpose of this study was to investigate the role of insulin resistance in HI. Although association of insulin resistance and PCOS is well defined, there are not enough data showing whether patients with HI also have insulin resistance. Therefore, we studied the presence of insulin resistance in PCOS, HI, and control groups. We found that patients with PCOS and HI have significantly higher insulin and HOMA-IR score than control subjects, whereas insulin level and HOMA-IR score were significantly lower in HI patients than in PCOS patients.

Hirsutism is a common endocrine disorder that represents increased androgens levels or androgen sensitivity. PCOS is the most common cause of hirsutism, and insulin resistance is well known as an important feature of PCOS [3]. On the other hand, hirsutism may be present without any diagnostic criteria for PCOS, as in women with HI.

The prevalence of HI has been reported as 13% in Iran [17] and 17.4% in Turkey [18]. Controversies exist concerning the presence of insulin resistance in HI. There are few studies that investigated the association of insulin resistance with HI. Some studies have shown that HI is not a separate entity from PCOS.

 Jahanphar and Eden conducted a study on 173 hirsute women and showed that the prevalence of PCOS in women with hirsutism is 86% and concluded that insulin resistance is a common finding in both PCOS and HI [19].

Similarly, Unluhizarci et al. studied 32 women with HI and 17 healthy women and concluded that HI is associated with some degrees of insulin resistance and suggested that insulin resistance and glucose intolerance may be associated with hirsute women regardless of whether they have PCOS or HI [14], that is, according to our results. 

Sheikholeslami et al. also showed that serum insulin level and HOMA-IR are significantly higher in PCOS group in comparison to HI group which is in consistent with our findings [20].

Fattah and Darwish concluded that there was a significant difference in the serum insulin level and HOMA-IR between PCOS, HI, and control groups, but in contrast with our results, there was not any difference in respect to insulin resistance between PCOS and HI groups [21]. Sarac et al. found that insulin-mediated glucose uptake was lower in HI group than in control group [22]. Ucak et al. demonstrated that nonobese HI patients also had insulin resistance [23]. Paoletti et al. showed that metformin (insulin-lowering drug) was effective on hyperinsulinemia and insulin resistance in patients with HI [24].

Since our groups contained BMI matched women, insulin resistance in HI patients cannot only be attributed to obesity. 

The relationship between insulin resistance and HI has not been explained well. One explanation might be the effect of insulin/insulin-like growth factor (IGF) system on hair follicle growth [25]. Weger and Schlake showed that IGF-1 has an important mitogenic effect on hair follicle in mice [26]. Thus, hyperinsulinemia raises the circulating IGF-1. This in turn results in cell growth in pilosebaceous unit [27].

Other explanations for the pathogenesis of HI include increased conversion of testosterone to dihydrotestosterone in hair follicles as a result of increased 5-*α* reductase activity [11], androgen receptor gene polymorphism [12], and also the increased sensitivity of pilosebaceous unit to the circulating androgens [13].

There are some studies that do not support an association between insulin resistance and HI. For example, Cebeci concluded that insulin level and insulin-resistant state of women with HI and control groups did not show any differences. This might be due to the small number of women with HI [28].

In conclusion, considering the above findings, it seems that hyperinsulinemia and insulin resistance are associated with hirsutism in both PCOS and HI. It seems that it is time to perform a meta-analysis study to explain the insulin resistance state in HI.

## Figures and Tables

**Table 1:** Baseline characteristics of three groups (PCOS, HI, and control) (mean ± SD).

Variable	Group			
	PCOS	HI	Control	*P* value
Weight (Kg)	66.5 ± 15.8	65.35 ± 11.2	65.4 ± 15.2	0.1
Height (cm)	159.8 ± 5.2	160.5 ± 5	160.2 ± 5.1	0.5
Age (year)	26.1 ± 5.3	26.9 ± 5.5	26.5 ± 5.4	0.5
BMI (Kg/M^2^)	25.4 ± 3.7	25.9 ± 3.1	25.1 ± 3.5	0.1

**Table 2:** Prevalence of symptoms in two groups of hirsutism (PCOS and HI) (number/%).

Variable	Group				
	PCOS		HI		*P* value
	*n*	%	*n*	%	
Galactorrhea	2	3.3	0	0	0.5
Normal mense	11	18.3	30	100	0.02
Acne	28	46.7	6	20	0.02
Hair loss	31	51.7	14	46.7	0.8
Infertility	6	10	1	3.3	0.4
Family history	40	66.7	24	80	0.2

**Table 3:** Baseline lipids levels in three groups (PCOS, HI, and control) (mean ± SD).

Variable	Group			
	PCOS	HI	Control	*P* value
TG (mg/dL)	140.6 ± 70.6	137.4 ± 33.1	140.4 ± 35.1	0.6
COL (mg/dL)	185.2 ± 33.6	190 ± 56.7	190.3 ± 45.1	0.6
HDL (mg/dL)	43.37 ± 7.2	42.07 ± 5.5	43.1 ± 5.3	0.8
LDL (mg/dL)	115.7 ± 24	123.2 ± 35	120.3 ± 27	0.5

**Table 4:** Baseline hormonal parameters in three groups (PCOS, HI, and control) (mean ± SD).

Variable	Groups			
	PCO	HI	Control	*P* value
Testosterone (ng/mL) (0.1–0.9)	0.89 ± 0.1	0.7 ± 0.1	0.6 ± 0.05	0.7
DHEA-S (ng/mL) (900–3600)	2414 ± 120	1823 ± 142	1240 ± 131	0.001*
Androstenedione (ng/mL) (0.75–3.2)	1.7 ± 0.4	1.69 ± 0.5	1.5 ± 0.4	0.3
17OHP (ng/mL) (0.35–2.90)	1.7 ± 1.2	1.4 ± 0.4	1.5 ± 0.2	0.4
Prolactin (ng/mL) (5–35)	22.8 ± 12.1	22.01 ± 2.8	22.1 ± 2.2	0.7
Insulin (*µ*Iu/mL) (2–20)	16.04 ± 1.4	7.32 ± 6.8	4.2 ± 1.8	0.001
FPG (mg/dL) (75–100)	91.8 ± 10.2	89.3 ± 9.4	88.9 ± 10	0.2
HOMA-IR	3.7 ± 3.3	1.7 ± 1.6	1.1 ± 0.9	0.001
Free testosterone (pg/mL)	3.2 ± 0.1	2.2 ± 0.1	1.6 ± 0.1	0.05

*Comparison of variables between three groups was made by Kruskal-Wallis test, and then comparison of DHEA-S between each couple of them was made by Students, *t*-test or Mann-Whitney *U* test.
